# The RING-CH Ligase K5 Antagonizes Restriction of KSHV and HIV-1 Particle Release by Mediating Ubiquitin-Dependent Endosomal Degradation of Tetherin

**DOI:** 10.1371/journal.ppat.1000843

**Published:** 2010-04-15

**Authors:** Claire Pardieu, Raphaël Vigan, Sam J. Wilson, Alessandra Calvi, Trinity Zang, Paul Bieniasz, Paul Kellam, Greg J. Towers, Stuart J. D. Neil

**Affiliations:** 1 MRC Centre for Medical Molecular Virology, University College London, London, United Kingdom; 2 Department of Infectious Disease, King's College London School of Medicine, Guy's Hospital, London, United Kingdom; 3 Howard Hughes Medical Institute, Aaron Diamond AIDS Research Center and Laboratory of Retrovirology, The Rockefeller University, New York, New York, United States of America; 4 Wellcome Trust Sanger Institute, Hinxton, Cambridge, United Kingdom; University of Southern California School of Medicine, United States of America

## Abstract

Tetherin (CD317/BST2) is an interferon-induced membrane protein that inhibits the release of diverse enveloped viral particles. Several mammalian viruses have evolved countermeasures that inactivate tetherin, with the prototype being the HIV-1 Vpu protein. Here we show that the human herpesvirus Kaposi's sarcoma-associated herpesvirus (KSHV) is sensitive to tetherin restriction and its activity is counteracted by the KSHV encoded RING-CH E3 ubiquitin ligase K5. Tetherin expression in KSHV-infected cells inhibits viral particle release, as does depletion of K5 protein using RNA interference. K5 induces a species-specific downregulation of human tetherin from the cell surface followed by its endosomal degradation. We show that K5 targets a single lysine (K18) in the cytoplasmic tail of tetherin for ubiquitination, leading to relocalization of tetherin to CD63-positive endosomal compartments. Tetherin degradation is dependent on ESCRT-mediated endosomal sorting, but does not require a tyrosine-based sorting signal in the tetherin cytoplasmic tail. Importantly, we also show that the ability of K5 to substitute for Vpu in HIV-1 release is entirely dependent on K18 and the RING-CH domain of K5. By contrast, while Vpu induces ubiquitination of tetherin cytoplasmic tail lysine residues, mutation of these positions has no effect on its antagonism of tetherin function, and residual tetherin is associated with the trans-Golgi network (TGN) in Vpu-expressing cells. Taken together our results demonstrate that K5 is a mechanistically distinct viral countermeasure to tetherin-mediated restriction, and that herpesvirus particle release is sensitive to this mode of antiviral inhibition.

## Author Summary

To replicate efficiently in their hosts, viruses must avoid antiviral cellular defenses that comprise part of the innate immune system. Tetherin, an antiviral membrane protein that inhibits the release of several enveloped viruses from infected cells, is antagonized by the HIV-1 Vpu protein. The K5 protein of the human pathogen Kaposi's sarcoma-associated herpesvirus (KSHV) modulates the cell surface levels of several host proteins including tetherin. We show that KSHV release is sensitive to tetherin, and that K5 expression is required for efficient virus production in tetherin-expressing cells. K5 is also capable of rescuing Vpu-defective HIV-1 virus release from tetherin. K5 expression induces a down-regulation of cell-surface tetherin levels and degradation in late endosomes, which depends on a single lysine residue in the tetherin cytoplasmic tail. Finally, we show that the ESCRT pathway, which promotes the trafficking of cell surface receptors for degradation, is required for K5-mediated tetherin removal from the plasma membrane. Thus, we demonstrate that herpesviruses are sensitive to the antiviral effects of tetherin and that KSHV has evolved a mechanism for its destruction. These findings extend the list of viruses sensitive to tetherin, suggesting that tetherin counter-measures are widespread defense mechanisms amongst enveloped viruses.

## Introduction

The inhibitory effect of type 1 interferons (type 1 IFN) on the replication of mammalian viruses has been documented for over 50 years. However the effecter mechanisms that interfere with virus replication have not been well characterized. While many IFN response genes are known, few definitive antiviral functions have been ascribed to them. Amongst the best characterized are PKR/2′5′oligoadenylate synthetase, MxA and ISG15, all of which have broad activity against a variety of mammalian RNA viruses [1]. Recently the identification of retroviral restriction factors including members of the APOBEC3 family of cytidine deaminases, as well as TRIM5 and other members of the tripartite motif protein family, has highlighted innate intracellular defense mechanisms as key determinants of tropism for human and primate immunodeficiency viruses [2], [3]. Moreover, these antiviral activities have driven the acquisition of viral countermeasures [2], [4] and thus interferon-inducible restriction factors are now thought to represent an important arm of the antiviral innate immune system [3].

Tetherin, (BST2/CD317) has recently been shown to inhibit the release of HIV-1 particles that are defective for the accessory protein Vpu [5], [6]. In the absence of Vpu expression, nascent HIV-1 particles assemble at the plasma membrane but remain tethered to the surface of tetherin expressing cells via a protease-sensitive linkage. Tethered virions are then endocytosed leading to their accumulation in late endosomes [5], [7], [8]. Tetherin colocalization with restricted viral particles on cell surfaces and in endosomes is well documented [5], [6], [9]. Strikingly, it is tetherin's unusual topology that is thought to be directly responsible for its mode of action [10]. Tetherin is a dimeric type-II membrane protein consisting of an N-terminal cytoplasmic tail, an extracellular domain with a putative coiled coil, and a C-terminal GPI anchor which is required for its antiviral function [5], [11]. It forms dimers which are thought to cross-link viral and cellular membranes during viral budding [10]. Tetherin appears to have no direct association with any viral structural proteins and is therefore able to restrict a range of unrelated viruses including retroviruses, filoviruses and arenaviruses [9], [12], [13]. It is expressed on mature B cells and plasmacytoid dendritic cells, but can be induced in many cell types by type-I interferon (IFN) [8], [14], [15], [16], [17]. Sequence analysis of orthologues of tetherin from primates indicates high levels of positive selection during their evolution suggesting selective pressure from pathogenic viral infections [18], [19]. Together, these observations suggest that tetherin may be an important antiviral defense against enveloped virus replication, necessitating the acquisition of viral countermeasures to antagonize its activity.

Interestingly, other potential viral countermeasures to tetherin may exist. Kaposi's sarcoma-associated herpesvirus (KSHV), also known as Human herpesvirus 8 (HHV8), encodes two immuno-modulatory membrane associated RING-CH (MARCH) E3 ubiquitin ligases named K3 and K5 [20]. K5 has been shown to mediate the down-regulation of a variety of cell surface markers including MHC class I, PE-CAM-1, CD80/CD86, ICAM-1, IFNγ receptor and NKG2D [21], [22], [23], [24], [25], [26], [27]. In a recent proteomics screen Bartee and colleagues used a methodology called stable isotope labeling of amino acids in cultured cells (SILAC) to identify proteins that are removed from the plasma membrane on K5 expression. One of the novel K5 targets found was tetherin (BST2) [28]. Here we have tested the hypothesis that tetherin restricts assembly/release of KSHV particles and that an important function of K5 is to overcome this process.

## Results

### KSHV particle release is sensitive to tetherin-mediated restriction

Tetherin has the capacity to restrict the release of diverse enveloped viruses including filoviruses and arenaviruses [9], [12], [13]. Given the capacity of the KSHV protein K5 to reduce cell surface expression of tetherin [28] we tested whether KSHV could be restricted by the expression of increasing amounts of exogenous human tetherin. We reasoned that the complex envelopment strategy of herpesviruses [29] and the tropism of KSHV for tetherin-positive mature B cells made this virus potentially sensitive to restriction by tetherin. To test this we generated a HeLa cell line harboring latent recombinant rKSHV.219 episomes under puromycin selection which encode eGFP driven by the human EF-1α promoter and DsRed driven by the KSHV PAN promoter that responds to the KSHV immediate early protein RTA [30]. Transfection of these cells with an RTA encoding plasmid (pCMV RTA) induces KSHV transcription, lytic replication and the release of KSHV particles, the efficiency of which can be assessed by measurement of RFP expression. The amount of virus released 48h post-RTA induction can be measured by quantitative PCR (Q-PCR) detecting DNAse-I protected genomes in the supernatant or by titration of infectious virus onto 293T cells and enumeration of GFP expressing cells by flow cytometry.

**Figure 1:**
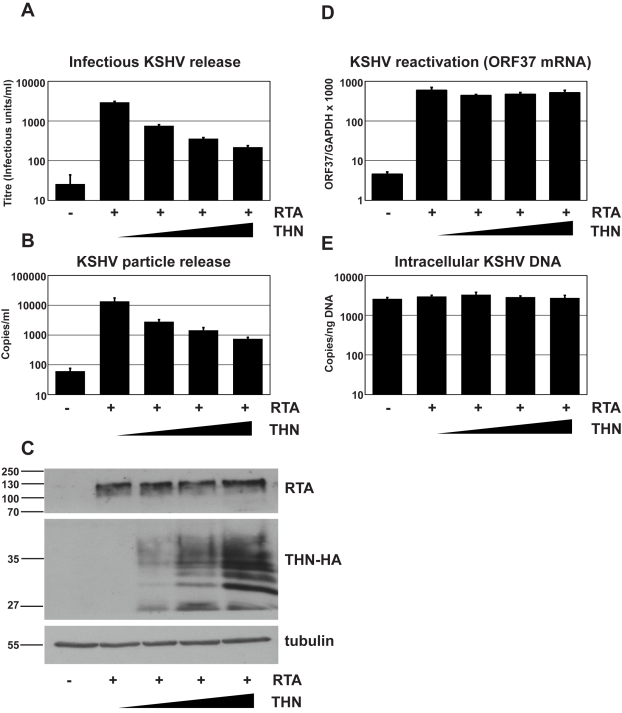
Over-expression of human tetherin restricts KSHV particle release. HeLa KSHV cells were transfected with an expression vector encoding the KSHV early transcriptional activator RTA and increasing doses of pCR3.1 encoding human tetherin. Plasmid dose was kept constant using pcDNA3.1. 48 hours after transfection supernatants were harvested, filtered and used to infect 293T cells (**A**) and infectious virus titer determined by GFP expression in the target cells by flow cytometry. Values are presented as infectious units/ml. (**B**) Parallel supernatants were treated for 2 hours with DNase-I and viral genomic DNA isolated and supernatant genome copy number enumerated by quantitative Taqman PCR specific for ORF37. Values are presented as copies/ml. (**C**) Western blot analysis for RTA protein (top panel) performed on r219-HeLa cells after virus collection indicates that RTA expression levels were equivalent in each sample. Blots were stripped and detection of tetherin using an anti-HA antibody performed (middle panel) shows increasing tetherin levels across samples, as expected. Alpha tubulin was detected concomitantly to RTA to demonstrate equal loading (bottom panel). (**D**) KSHV reactivation was equivalent in all samples as evidenced by measurement of ORF37 mRNA levels by quantitative Taqman PCR and after normalization to GAPDH levels. (**E**) KSHV genome levels remained constant in all samples as evidenced by equivalent numbers of intracellular KSHV episomes per nanogram of total cellular DNA. Results are the mean of 2 independent experiments and errors are standard error of the mean.

We first determined whether transfecting a plasmid expressing tetherin together with pCMV-RTA would impact on the amount of infectious virions released into the supernatant. Figure 1A clearly demonstrates a linear decrease in release of KSHV infectious virus when increasing amounts of the tetherin-expressing plasmid are transfected with a constant amount of pCMV-RTA. Similarly, Q-PCR performed on DNAse-I resistant genomes showed a greater than 10-fold decrease in total virions released for the highest dose of tetherin-expressing plasmid used (Figure 1B). To control for transfection efficiency, cells were recovered after virus collection and cell lysates subjected to western blotting (Figure 1C). As expected, increasing amounts of tetherin were detected as increasing amounts of tetherin-expressing plasmid was transfected. Importantly, equal RTA expression was found in all samples confirming that RTA levels were not impacted by tetherin expression. To further rule out an effect of tetherin expression on KSHV reactivation we also measured lytic viral RNA production by quantitative RT-PCR. Ct values for ORF37 were normalized to those obtained for cellular GAPDH. Figure 1D shows very similar ORF37/GAPDH ratios for each condition. Whilst unlikely, it is also possible that exogenous expression of increasing amounts of tetherin might lower genomic replication, thus leading to a reduced amount of virions and/or DNase-I resistant genomes in the supernatant. To rule this out we measured intracellular episomes in DNA extracts by Q-PCR. Figure 1E clearly demonstrates that episome copy number was similar across all conditions and could not account for reduced viral production.

### Reduction of K5 expression using K5 specific shRNA reduces KSHV release

**Figure 2:**
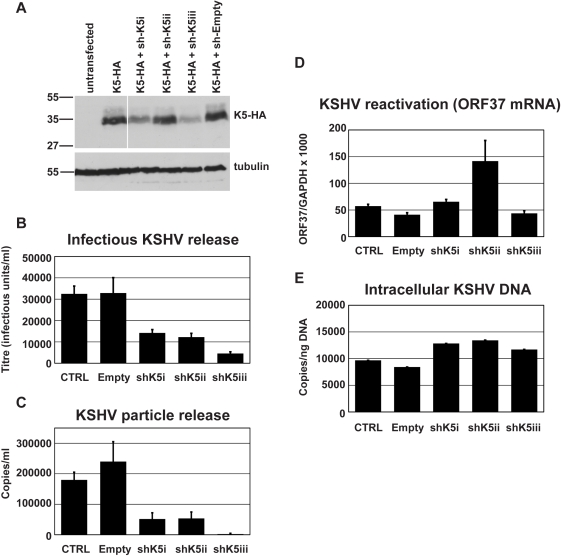
RNAi-depletion of K5 during lytic replication suppresses KSHV particle release. (**A**) Three shRNA hairpins were designed to target K5 and co-expressed with K5-HA in 293T cells. The efficiency of reduction of K5 expression was assessed 48 hours later by western blot of total cell lysates detecting the HA tag. Stripped blots were re-probed for alpha tubulin to demonstrate equal loading. (**B & C**) r219-HeLa cells were transduced by lentiviral vectors encoding K5 shRNAs at an MOI of 5, reseeded 72 hours later and transfected with RTA expression plasmid the following day. Supernatants were harvested 48 hours post-RTA transfection and KSHV titer expressed as infectious unit/ml (**B**) and DNase-I resistant genome copies/ml (**C**) were determined as in Figure 1. At the time of harvest (48 hours post RTA transfection), ORF37 and GAPDH mRNA were measured in the cell lysates by Taqman Q-RT-PCR to assess effect of the hairpins on KSHV reactivation and ORF37 expression (**D**). At the time of harvest, cells were also kept to assess the effect of the hairpins on genome replication. Cellular DNA was extracted and KSHV episomes measured by QPCR for ORF37 (**E**). Results are expressed as KSHV genomes per nanogram of total cellular DNA. Results are the mean of 2 independent experiments and errors are standard error of the mean.

We then tested whether K5 expression was required for efficient KSHV release from r219-HeLa cells. We reasoned that if K5 is a KSHV antagonist for tetherin then reducing its expression by RNA interference (RNAi) should inhibit KSHV release. Three shRNA hairpins were designed and expressed in 293T cells together with a HA-tagged K5 protein. Reduction of K5 expression by the hairpins was assessed by recovering the cells 48 hours after transfection and subjecting the lysates to western blot (Figure 2A). Blots probed with the anti-HA antibody were stripped and re-probed for alpha tubulin to control for equal loading (Figure 2A). All three hairpins reduced K5 expression with hairpin 3 (sh-K5iii) being the most potent. We then expressed the hairpins in r219-HeLa cells using lentiviral vectors [31]. 72 hours later the cells were re-seeded and transfected with RTA to induce KSHV lytic replication. Release of infectious KSHV was measured by titration of supernatants on 293T as before (Figure 2B). We found that expression of specific anti-K5 shRNA reduced KSHV titer in the supernatant whereas expression of an empty shRNA vector did not (Figure 2B). The number of DNAse-I resistant KSHV genomes in the supernatant was also reduced by all three shRNA vectors, as shown by taqman Q-PCR (Figure 2C). Messenger RNA for the late gene ORF37 was measured by quantitative RT-PCR in each sample and values were corrected for cellular GAPDH mRNA levels. This demonstrated that hairpin expression did not significantly inhibit KSHV reactivation (Figure 2D). In fact hairpin 2 appeared to stimulate ORF37 expression in one experiment leading to the large error bar. However, this experiment shows that inhibition of reactivation cannot account for the loss of KSHV in the supernatant. To further rule out an effect of hairpin expression on genomic replication we also measured intracellular KSHV episomes by DNA Q-PCR for ORF37 (Figure 2E) This control confirmed that the number of KSHV episomes in the cells did not account for the K5 hairpin induced defect in KSHV release and supports the notion that K5 antagonizes tetherin.

These results suggest that K5 is required for efficient KSHV particle release in a cell line that constitutively expresses tetherin and that expressing increasing amounts of exogenous tetherin further inhibits KSHV release. Together our data suggest that tetherin has antiviral activity against KSHV and that K5 has an important role in overcoming tetherin-mediated restriction.

### K5 can substitute for Vpu in rescuing HIV-1 particle release from tetherin-mediated restriction

**Figure 3:**
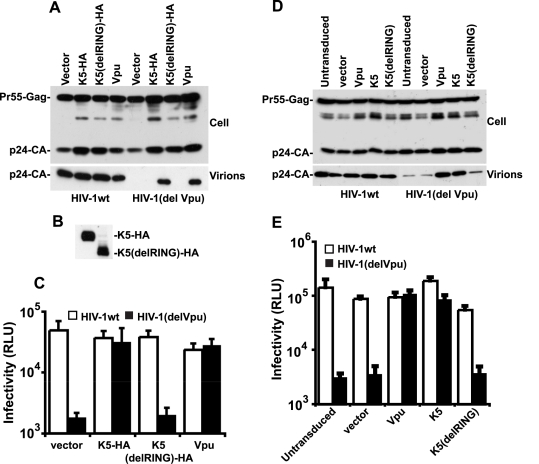
K5 rescues Vpu-defective HIV-1 particle release from tetherin mediated restriction. (**A, B and C**) HeLa cells were co-transfected with HIV-1wt or HIV-1(del Vpu) proviral plasmids in combination with empty vector, or expression vector encoding Vpu, HA-tagged K5 or K5 lacking the N-terminal RING domain. 48h post-transfection cell lysates and pelleted supernatants were analysed for HIV-1 Gag proteins by western blot using an anti-p24CA monoclonal antibody (**A**) or an anti-HA monoclonal (**B**). Viral supernatants were also used to infect HeLa-TZM indicator cells and infectious viral release determined by relative beta-galactosidase activity 48h later (**C**). (**D and E**) HT1080 cells stably expressing human tetherin (HT1080/THN-HA) were transduced with retroviral vectors encoding both dsRED and either Vpu, K5 or K5(del RING) at doses sufficient to give >90% transduction as determined by flow cytometry. Cells were then infected with HIV-1wt or HIV-1(del Vpu) pseudotyped with VSV-G at an MOI of 0.2. Cell lysates and supernatants were processed as in (**A**) 48h later.

Having established a role for K5 in counteracting tetherin during KSHV release we then tested whether K5 expression could functionally substitute for the HIV-1 encoded tetherin antagonist Vpu and rescue the release of tetherin restricted HIV-1(delVpu) particles [5]. We transfected HeLa cells with the HIV-1 molecular clone NL4.3, or a Vpu-defective counterpart, together with expression vectors for HA-tagged K5 or Vpu. As predicted, K5 expression potently rescued HIV-1(delVpu) release to levels achieved by Vpu expression (Figure 3A). This was evidenced both by measurement of HIV-1 p24 capsid protein released into the supernatant by western blots as well as by titration of infectious HIV-1 virus released into the supernatant on sensitive indicator cells (Figure 3A, B and C). Importantly K5 expression had no effect on wild-type HIV-1 particle release or on HIV-1 structural protein expression. These data demonstrate that K5 is a functional homolog of HIV-1 Vpu and that KSHV encodes a tetherin countermeasure.

The fact that K5 has been demonstrated to be a RING dependent E3 ligase for ubiquitin [20] suggested that the RING-CH domain is important for its function. We therefore made a RING deletion mutant of K5 (K5delRING) and tested its ability to counteract tetherin restriction in an HIV-1 release assay as above. Despite similar expression levels of the mutant and wild type K5 proteins (Figure 3B), the RING-defective K5 was unable to rescue HIV-1(del Vpu) release from HeLa cells (Figure 3A and C). To confirm that K5 can antagonize tetherin function we repeated the experiment but in a tetherin-deficient cell line (HT1080) [5] stably expressing a tetherin protein in which an HA-tag had been inserted into the ectodomain at amino acid position 154 [9]. HT/THN-HA cells were then transduced with retroviral vectors co-expressing DsRed and Vpu, or K5 or K5delRING at doses sufficient to give >90% DsRED-positive cells as demonstrated by flow cytometry 48 hours later. The cells were then re-seeded and infected with VSV-G pseudotyped HIV-1(wt) or HIV-1(del Vpu) at a multiplicity of infection of 0.2. Since HT1080 cells are devoid of CD4, VSV-G pseudotyping allows the measurement of a single round of viral replication. Similar to the experiment in HeLa cells, HT/THN-HA released HIV-1(del Vpu) particles approximately 20 fold less efficiently than they released wild type virus. Furthermore, expression of either K5 or Vpu, but not K5delRING, could rescue the tetherin mediated defect in virus release (Figure 3D and E). Measurement of gag levels in supernatants and extracts of infected cells by western blot indicated that the effects of tetherin, Vpu and K5 were on HIV-1 release and not gag protein expression. Intriguingly, K5 could not antagonize tetherin function in transiently-transfected 293T cells, even when tetherin expression was titrated to vary its expression level (Figure S1). We propose that 293T cells lack a co-factor essential for K5's antagonism of tetherin, but not for Vpu function. Interestingly, 293T cells are also unable to support HIV-2 Env's anti-tetherin activity [32], suggesting that this particular cell line might be poorly suited for characterization of some tetherin antagonists.

### Species-specific down-regulation of cell surface tetherin by K5

**Figure 4:**
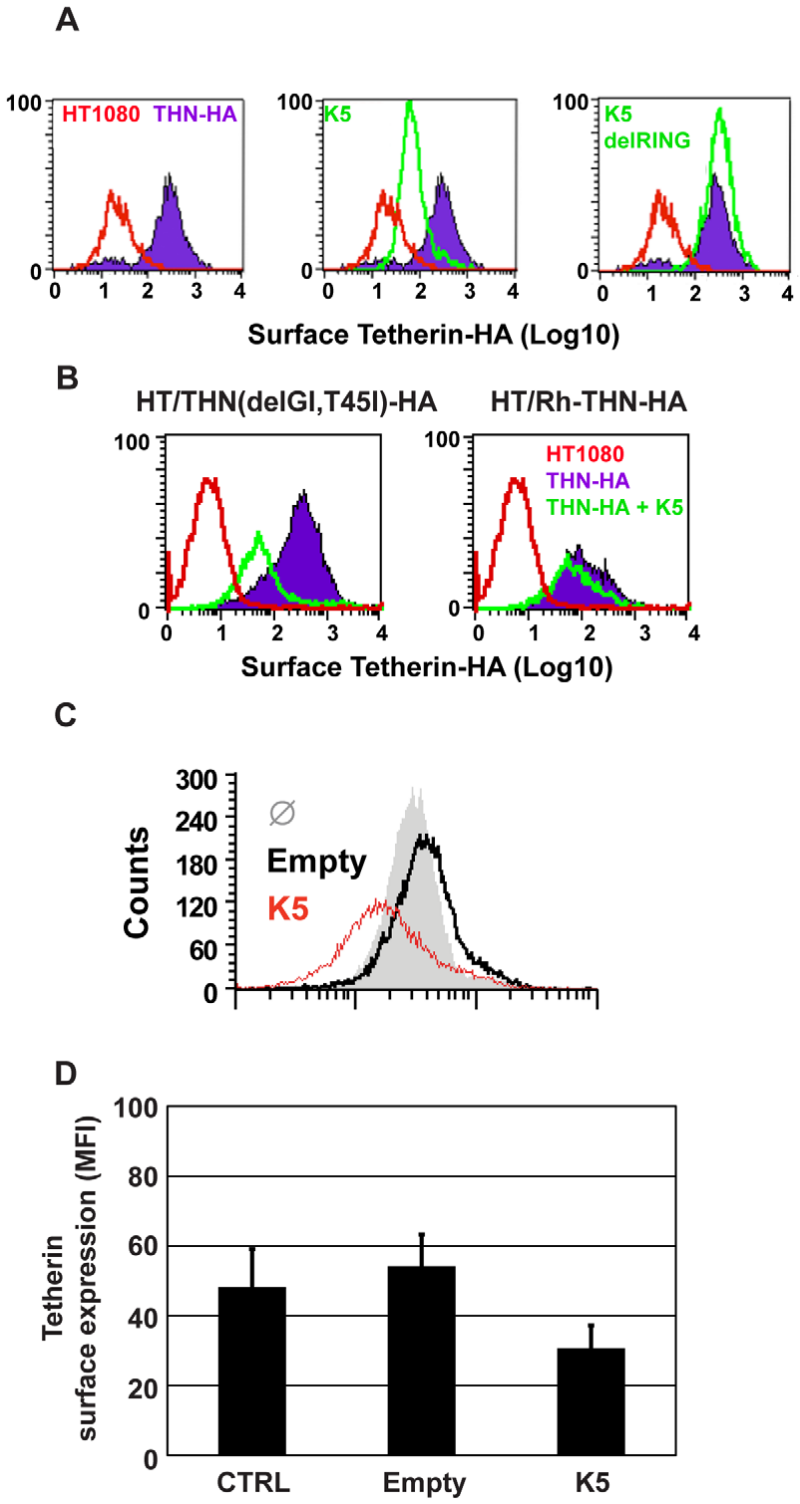
K5 mediates cell-surface down-regulation tetherin. (**A**) HT1080/THN-HA cells were transduced with retroviral vectors encoding dsRED and either K5 or K5(del RING). 48h post-transduction the cells were immunostained for surface tetherin using an anti-HA monoclonal antibody and an Alexa-488-conjugated goat-anti-mouse secondary antibody. Surface expression of tetherin was then analyzed in the dsRED positive population by flow cytometry. Parental HT1080 cells were used as a control for background antibody binding and are represented by the red line. Tetherin levels in HT1080 cells expressing tetherin alone are indicated by the purple line and levels of tetherin after K5 expression is represented by the green line. (**B**) The same analyses were performed on HT1080 cells expressing the HA-tagged Vpu-resistant human tetherin (delGI,T45I) and rhesus tetherin. Labeling is similar to panel A. (**C**) BCBL1 were transduced with a lentiviral vector expressing K5 (red line), an empty vector (black line), or left untreated (grey peak), and cell surface expression of tetherin detected by flow cytometry 48 hours later as described above. (**D**) Mean fluorescence intensity (MFI) values from (**C**) are also shown.

Vpu has been shown to remove tetherin from the cell surface after transfection [6] and in HIV-1-infected cells [32]. We therefore tested whether K5 expression had the same effect on surface tetherin levels in HT/THN-HA cells by expressing K5 via retroviral transduction. Both K5 (Figure 4A) and HIV-1 Vpu (Figure S2) expression led to a marked reduction of tetherin from the cell surface. Importantly, the K5delRING protein was inactive in this assay as predicted by data in Figure 3. HIV-1 Vpu-mediated tetherin antagonism displays distinct species specificity for primate tetherin genes with non-human primate orthologues being largely insensitive to HIV-1 Vpu [18], [19], [33]. Sensitivity to HIV-1 Vpu maps to the tetherin transmembrane domain and mutation of the T residue at position 45 to the I present in Rhesus macaques (T45I) coupled with an in frame deletion of a GI pair at the N-terminus of the human tetherin protein's TM (delGI-T45I) results in a human tetherin that is completely resistant to HIV-1 Vpu [18] (Figure S2). We therefore examined whether K5 also displays similar species–specific effects (Figure 4B). Unlike HIV-1 Vpu, K5 was able to down-regulate the THN(delGI-T45I) mutant human tetherin. However, K5 was unable to down-regulate the rhesus macaque tetherin protein. Thus K5 also displays species-specificity in its antagonism of primate tetherins but the determinants of this specificity are distinct from those of HIV-1 Vpu.

We then examined whether K5 could also down regulate human tetherin from the surface of a B cell line carrying KSHV (Figures 4C and D). Body-cavity-based lymphoma (BCBL) 1 cells were transduced with a lentiviral vector encoding K5 or with an empty vector and surface tetherin expression assessed by flow cytometry analysis, using a specific antibody against human tetherin. K5 expression led to a reduction of cell surface tetherin as compared to the levels on cells treated with the empty vector or levels detected on un-transduced cells (Figure 4C). The mean of mean fluorescence intensity (MFI) values for 3 representative experiments are also shown (Figure 4D).

### K5 induces ubiquitin-dependent endolysozomal degradation of tetherin

**Figure 5:**
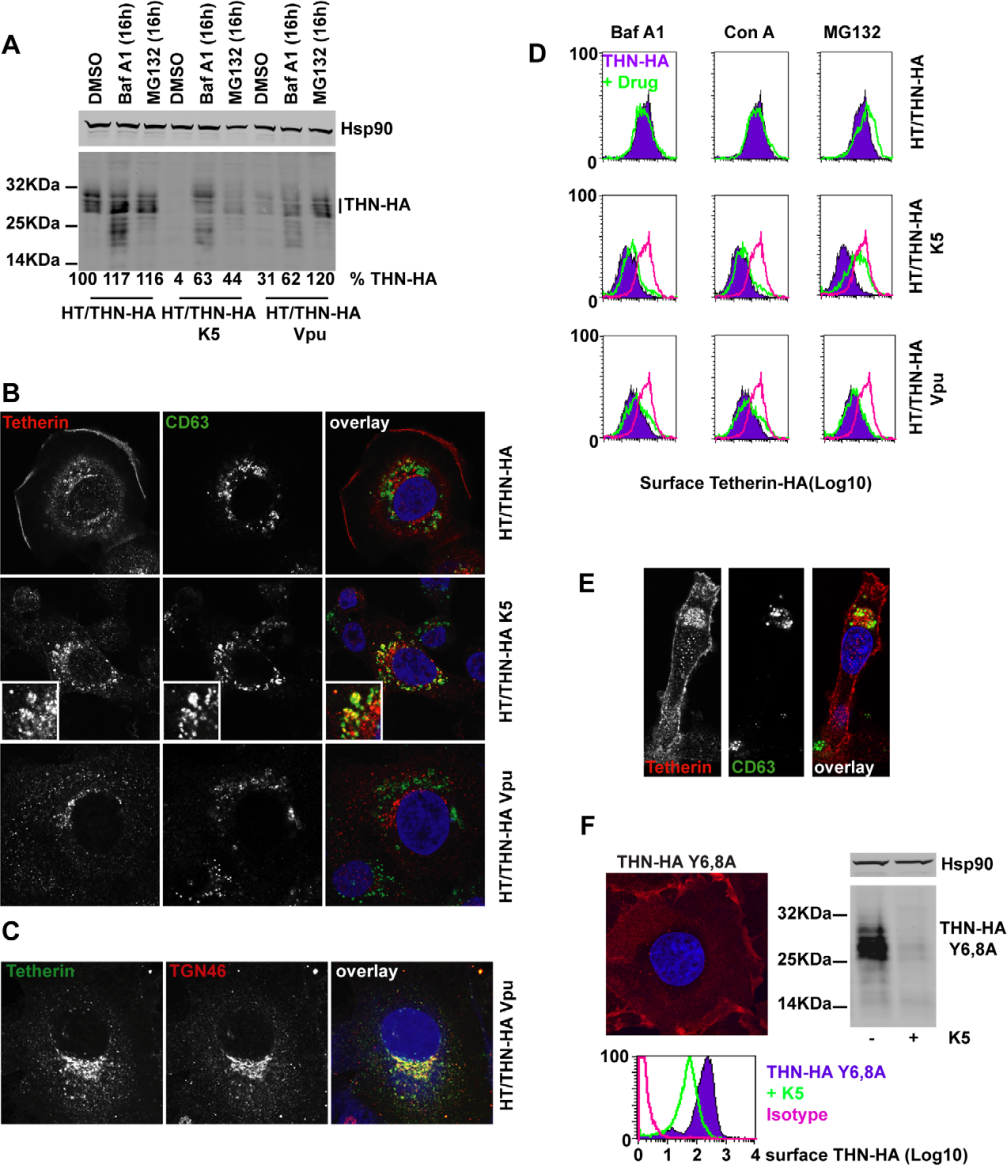
Effects of proteasomal and endosomal inhibitors on tetherin levels and localization in cells expressing K5 and Vpu. (**A**) Western blots of cell lysates from HT/THN-HA cells and isogenic pools stably expressing K5 or Vpu from retroviral vectors. Cells were treated for 16h with BafA1 (100nM), MG132 (1µg/ml) or DMSO as a control, lysed and separated by SDS-PAGE. THN-HA was detected by anti-HA polyclonal antibody, with Hsp90 serving as a loading control and visualized using Licor fluorescently coupled 650 and 800 nm secondary antibodies. Percent mature tetherin levels, normalized to Hsp90 loading are displayed below each lane. (**B**) Representative examples of HT/THN, HT/THN-HA K5 and HT/THN-HA Vpu cells immunostained for tetherin with a rabbit anti-HA antibody (red) and an antibody for the late endosomal marker CD63 (green). Nuclei were counterstained with DAPI (blue) and cells examined by confocal microscopy. (**C**) HT/THN-HA Vpu cells stained for THN-HA (green) and the trans-Golgi marker TGN46 (red) were processed as above. (**D**) Cells from A were surface stained for tetherin levels and analyzed by flow cytometry. Purple histograms represent tetherin levels on DMSO treated cells. Green overlays indicate tetherin levels after treatment with the indicated drug. The pink histogram overlays show the levels of tetherin on untreated HT/THN-HA from the upper row for comparison. (**E**) Confocal image of a representative HT/THN-HA K5 cell treated with MG132 and stained for THN-Ha (red) and CD63 (green). (**F**) HT/THN-HA Y6,8A were imaged by confocal microscopy for tetherin (red). The cells were then manipulated to express K5 and analyzed by flow cytometry for surface tetherin and by western blot for total cellular tetherin levels.

We next examined the fate of tetherin in K5-expressing cells. HT/THN-HA cells were transduced to stably express K5 or Vpu and intracellular levels of tetherin were compared to levels in unmanipulated cells. Tetherin appears in western blots as a heterogeneous smear of glycosylated species that varies between cell types [10], [17]. After K5 and Vpu expression, tetherin levels in the modified cell lysates were decreased (to 4% of wildtype in the case of K5 and to 31% of wildtype for Vpu), suggesting that, like Vpu, K5 induces tetherin degradation (Figure 5A). At present it is unclear whether Vpu induces tetherin degradation via a proteasomal-dependent [19], [34], [35] or lysozomal pathway [36], [37]. This is further complicated by the fact that endolysozomal degradative pathways are often also dependent on ubiquitin, and thus proteasomal inhibition can inhibit them through depletion of free cytoplasmic ubiquitin levels. We addressed the K5 mechanism of action by examining the role of lysozomal degradation using the vacuolar ATPase inhibitor bafilomycin A1 or by inhibiting proteasomal degradation using MG132. We then measured steady state levels of tetherin in K5 or Vpu expressing cells. A 16h treatment with BafA1 or MG132 substantially rescued tetherin levels from both Vpu and K5 expression. BafA1 treatment rescued not only mature tetherin species, but also lower molecular weight fragments that are likely to be partially degraded tetherin molecules. This suggested that K5 degrades tetherin via an endolysozomal process, similar to that by which it degrades Class I MHC [38]. The rescue of tetherin degradation products in HT/THN-HA cells that do not express HIV-1 Vpu or K5 suggested that endosomal processing of tetherin contributes to its natural turnover. In contrast, whilst MG132 treatment also rescued partially tetherin levels in HT/THN-HA-K5 cells, mature species were predominant. In Vpu-expressing cells MG132 appeared more potent for tetherin rescue than BafA1, and again differential tetherin species were rescued by each inhibitor. Together, these data suggest that while tetherin degradation by K5 and Vpu are sensitive to both classes of inhibitor, the stages of degradation that are affected are different.

Next we determined whether we could observe differences in the cellular localization of tetherin induced by K5 or Vpu. In HT/THN-HA cells, tetherin localized predominantly to the plasma membrane with a small amount of the intracellular localizations coincident with the late endosomal marker CD63 (Figure 5B) This is consistent with the notion of natural tetherin turnover in endolysozomal compartments. In K5 expressing cells, while tetherin levels are markedly reduced, remaining tetherin was much more often found associated with CD63+ late endosomes than in controls (Figure 5B), implicating K5-induced endosomal degradation. In contrast, in Vpu-expressing cells, tetherin was never observed in CD63+ endosomes, but instead localized predominantly to compartments that stain positive for the Trans-Golgi marker TGN46 (Figure 5B and C). This localization is similar to that observed after expression of the HIV-2 and SIVtan envelope glycoproteins [32], [39]. Thus, the subcellular localization of tetherin after K5 expression suggests that K5 causes it to be delivered to late endosomes for degradation. Importantly, this distinguishes K5 and Vpu-induced tetherin antagonism and implies distinct mechanisms.

Since the RING domain of K5 is required for tetherin down-regulation from the cell surface, and degradation is sensitive to the drug MG132, which also causes ubiquitin depletion, we hypothesized that K5 mediated ubiquitination might drive tetherin delivery to late endosomes. We therefore examined whether proteasomal or lysozomal inhibition could rescue cell-surface tetherin levels (Figure 5D). We found that whilst endosomal inhibition with BafA1 treatment rescued tetherin protein in the cell extracts of K5 expressing cells (Figure 5A) neither BafA1 or concanamycin A could rescue tetherin levels at the cell surface (Figure 5D). However, consistent with a role for ubiquitination in the delivery of tetherin for endosomal degradation, MG132 treatment of tetherin-expressing cells did substantially rescue tetherin levels at the surface of K5-expressing cells (Figure 5D and E). In contrast, none of the inhibitors significantly rescued tetherin surface expression in Vpu-expressing cells. These observations demonstrate that K5-induces an endosomal degradation of tetherin and suggest that an ubiquitin dependent process is required for its delivery into this pathway. Conversely, HIV-1 Vpu causes tetherin degradation by a distinct mechanism that is associated with its localization to the TGN (Figure 5B–C).

Next we asked whether the double-tyrosine based endocytic motif in the tetherin cytoplasmic tail was required for K5-mediated degradation. This motif binds the clathrin adaptors AP1 and AP2 and has been reported to be important for tetherin endocytosis and recycling [40]. HT1080 cells expressing THN-HA Y6,8A were generated and tetherin localization determined by immunofluorescence microscopy. As expected this mutant tetherin was found almost exclusively at the plasma membrane (Figure 5F). Interestingly, expression of K5 in these cells still led to significant tetherin down-regulation and degradation (Figure 5F) as demonstrated by flow cytometry and western blot. Thus tetherin trafficking to late endosomes induced by K5 is independent of its endocytic motif, suggesting that K5 targets tetherin for degradation via a pathway that is independent from its normal subcellular trafficking.

### Delivery to late endosomes and antagonism of antiviral activity by K5 is dependent on a single lysine residue in the tetherin cytoplasmic tail

**Figure 6:**
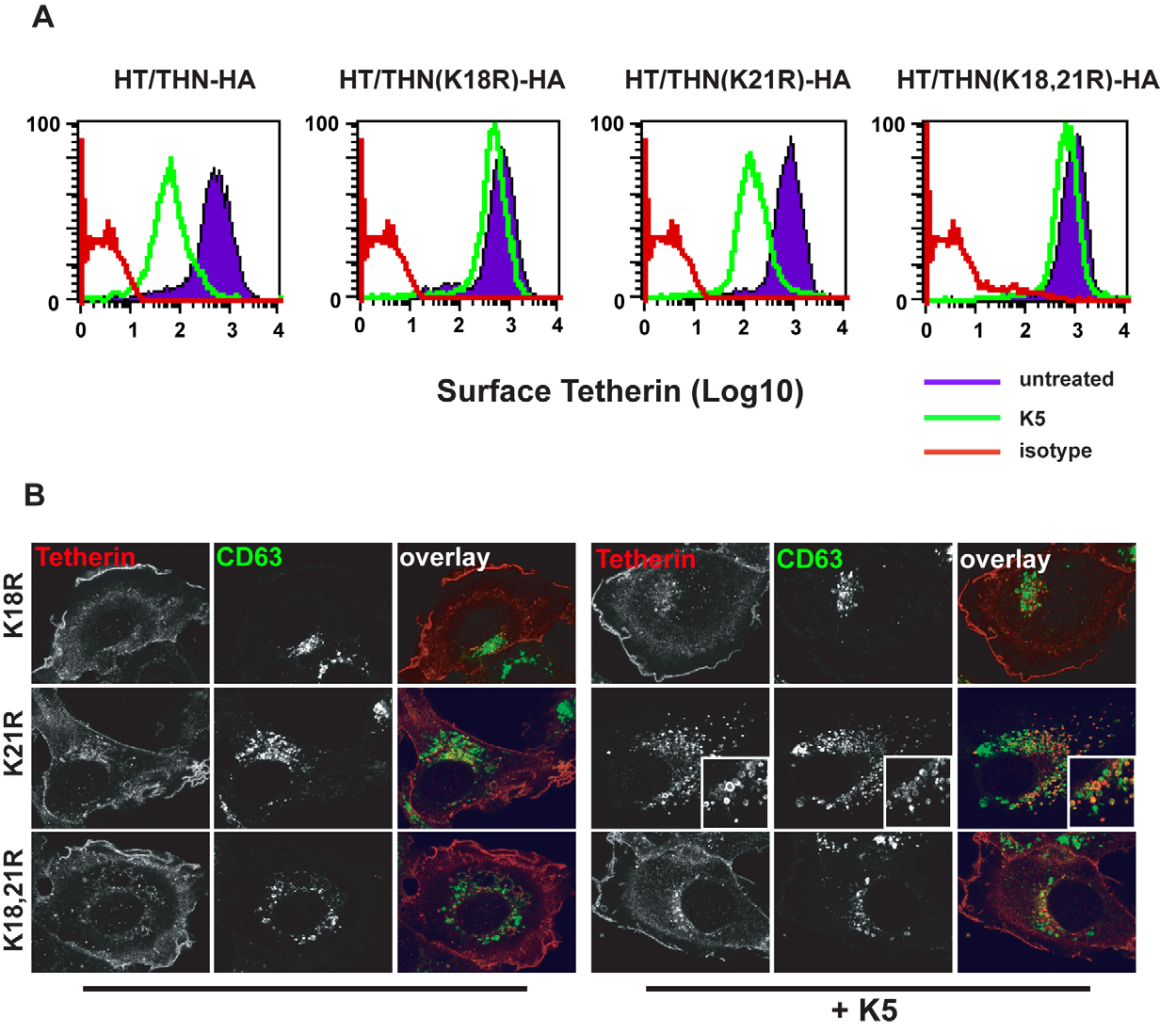
K18 in the tetherin cytoplasmic tail is required for K5-mediated cell surface down-regulation and delivery to endosomes. (**A**) Flow cytometry analyses of HT1080 cells expressing wild type tetherin or the indicated mutant. Purple histograms represent THN-HA levels on unmanipulated cells, with the green overlay showing tetherin levels in the equivalent cells stably expressing K5. Red histograms represent the antibody isotype control. (**B**) Representative examples the cells from (**A**) were stained for THN-HA (red) and CD63 (green) and examined by confocal microscopy.

**Figure 7:**
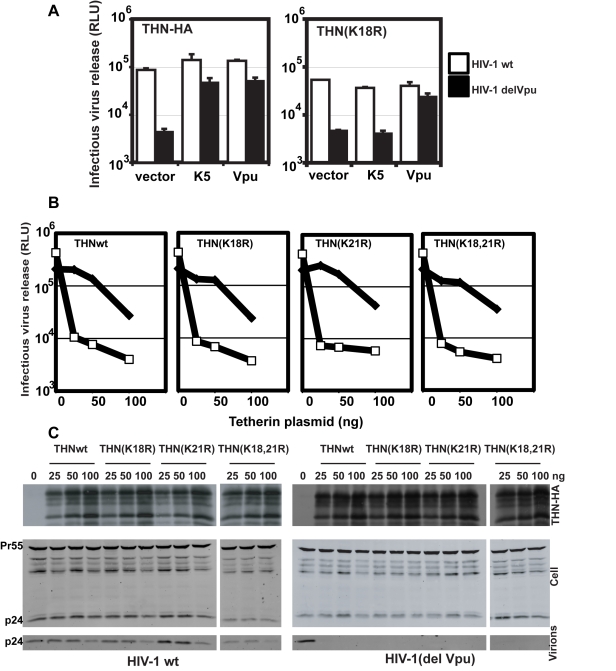
K18 is required for tetherin antagonism by K5 but both cytoplasmic tail lysines are dispensable for Vpu-mediated antagonism. (**A**) HT/THN-HA and HT/THN-HA(K18R) or derivatives stably expressing K5 or Vpu were infected with wild type HIV-1 or HIV-1(delVpu) VSV-G pseudotypes at an MOI of 0.2. 48h after infection cell supernatants were harvested and the released infectivity determined on HeLa-TZM cells. (**B and C**) The effect of tetherin lysine mutants on HIV-1 release in 293T cells. Cells were transiently transfected with wild type HIV-1 or HIV-1(delVpu) proviruses with increasing doses of the indicated THN-HA mutant. 48h after transfection viral supernatants were assayed for infectivity on HeLa-TZM cells (**B**) and cell lysates and pelleted virions analyzed by western blot for HIV-1 p24 CA and THN-HA expression (**C**).

K5 targeting of class I MHC molecules depends on membrane proximal lysine residues in their cytoplasmic tails [20]. Tetherin also has two membrane proximal lysines, K18 and K21. To seek a role for these residues we mutated them to arginine, singly or in combination, in THN/HA and stably expressed these mutant tetherins in HT1080 cells. We then stained the cell surface tetherin via the HA tag and measured cell surface tetherin levels by flow cytometry (Figure 6A). All tetherins were expressed at the cell surface, but the lysine mutants were expressed at enhanced levels as compared to the wild-type protein. This suggested that these two lysines might be involved in natural tetherin turnover. When K5 was expressed in the mutant tetherin cell lines, tetherin down-regulation was almost completely prevented for proteins bearing a K18R substitution. In contrast, K21R mutation had no effect on K5-induced cell surface down-regulation. Furthermore, K21R but not K18R or K18,21R mutants were consistently redistributed to CD63+ compartments upon K5 expression (Figure 6B). We then further examined whether K5 retained the ability to disrupt tetherin function in the absence of cell-surface tetherin down-regulation, as has been suggested for Vpu [17]. HT/THN-HA and HT/THN-HA K18R cells expressing either K5 or Vpu were infected with HIV-1wt and HIV-1(delVpu) VSV-G pseudotyped viruses at an MOI of 0.2. Supernatants were collected 48h later and infectious virus output was measured on HeLa-TZM cells. THN-HA K18R expressing cells restricted Vpu-defective HIV-1 release similarly to the wild type protein, however expression of K5 failed to rescue the release of HIV-1(delVpu) from HT/THN-HA-K18R cells (Figure 7A). By contrast Vpu-mediated antagonism of tetherin was unaffected by mutation of K18 in HT1080 cells (Figure 7A) or indeed either of the lysine residues in 293T cells (Figure 7B). Thus, removal of tetherin from the cell surface and delivery to late endosomes is required for K5-mediated antagonism of its antiviral action and this is dependent on the lysine at position 18. Measurement of gag levels in cell extracts and supernatants by western blot demonstrated that tetherin expression did not impact on gag expression (Figure 7C). Measurement of tetherin levels ensured that tetherin was expressed as expected (Figure 7C).

**Figure 8:**
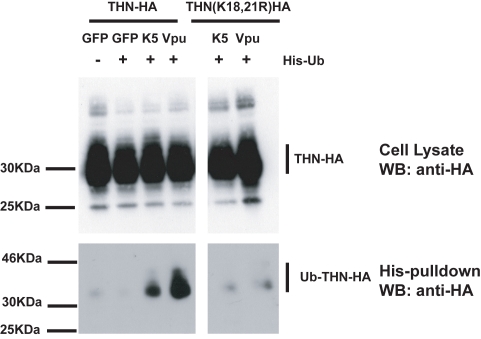
Tetherin cytoplasmic tail lysine residues are ubiquitinated in the presence of K5 and Vpu. HeLa cells were transiently transfected with the indicated THN-HA expression vector in combination with K5, Vpu or GFP and in the presence or absence of 6His-tagged ubiquitin. 48h after transfection, cells were treated for 8 hours with BafA1 (100nM) to prevent tetherin degradation. Cell lysates were then harvested and ubiquitinated proteins were isolated by binding to Ni-Nti-agarose. Cell lysates and pull-downs were analyzed by western blot for THN-HA detecting the HA tag.

Since lysine residues serve as targets for ubiquitination we next sought evidence for tetherin ubiquitination in the presence of K5. HeLa cells were transfected with THN-HA or THN-HA K18, 21R in the presence of an ubiquitin bearing a 6-histidine tag. The cells were treated for 8h with BafA1 to block tetherin degradation and ubiquitinated proteins were isolated by incubating whole cell lysates with nickel-agarose beads. In the presence of either co-transfected K5 or Vpu, THN-HA molecules could be isolated from the transfected cells (Figure 8). The tetherin predominantly formed a single species at a size suggestive of mono-ubiquitination. Interestingly, we failed to pull down ubiquitinated tetherin molecules when their cytoplasmic lysine residues had been mutated, after either K5 or Vpu transfection. This implies that the action of both K5 and Vpu leads to ubiquitination of the tetherin cytoplasmic tail. In the case of K5, this suggests that ubiquitination of K18 antagonizes tetherin-mediated restriction and directs it to endosomal compartments for degradation. Intriguingly, mutating the lysine residues does not make tetherin insensitive to Vpu suggesting that tetherin becomes ubiquitinated as a consequence of tetherin antagonism by HIV-1 Vpu but that this ubiquitination is not required for the antagonistic process.

### K5-mediated down-regulation of tetherin is sensitive to inhibition of the ESCRT pathway

**Figure 9:**
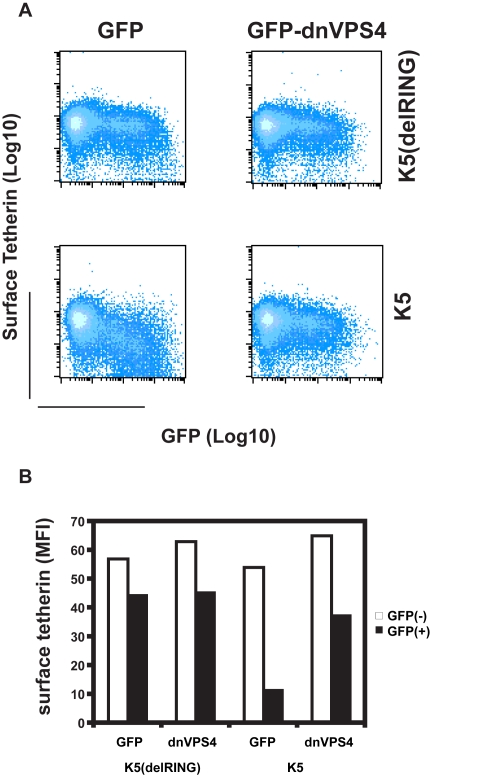
Cell-surface down-regulation of tetherin by K5 is inhibited by dominant-negative VPS4. (**A**) HeLa cells were transfected with wild type K5 or K5(del RING) expression vectors in combination with either GFP or a dominant negative mutant of VPS4 fused to GFP (VPS4 E228Q). 24h later cells were stained for cell surface tetherin using anti-BST2 monoclonal antibody and a goat-anti-mouse Alexa633 secondary antibody. Flow cytometry dot plots are shown. (**B**) The mean florescent intensities (MFI) of surface tetherin expression in GFP positive and GFP negative populations from the samples in panel A are plotted.

There are several ubiquitin-dependent mechanisms by which K5 might achieve tetherin degradation. For example, ubiquitination of tetherin's cytoplasmic tail could stimulate its internalization and mediate classical recruitment of the ESCRT pathway through engagement of TSG101, as has been shown for K3 targeting of MHC I [41]. This would lead to the budding of tetherin containing vesicles into the lumen of multivesicular bodies for degradation in lysozomes [42]. To examine the role of the ESCRT pathway in K5-mediated tetherin degradation, we tested whether K5-mediated loss of tetherin from the cell surface was sensitive to expression of a dominant negative form of VPS4. VPS4 is the essential AAA-ATPase that provides the energy for ESCRT disassembly and recycling during the final membrane scission event in the sorting of cell surface receptors for endosomal degradation [43]. Co-transfection of HeLa cells with GFP-dnVPS4(E228Q) [44] substantially rescued cell surface tetherin levels from K5 as assessed by flow-cytometry (Figure 9A and B). Expression of K5delRING has no effect on THN cell surface expression, concordant with Figure 4, and neither does the dominant negative VPS4 protein when expressed with the tetherin RING mutant. Together with the demonstration that K5 leads to tetherin ubiquitination and degradation, these observations strongly suggest that K5 induces a VPS4 and ubiquitination-dependent trafficking of tetherin from the cell surface to late endosomes for destruction. Importantly, this mechanism is similar to that used by K3 to down-regulate Class I MHC molecules [38], [41].

## Discussion

Tetherin has emerged as a potent inhibitor of enveloped virus release [5], [6], [9], [12], [13]. Recent evidence has demonstrated that tetherin dimers act as a physical linkage between the membranes of the infected cell and nascent virions [10]. This mechanism lends itself well to a general non-specific antiviral inhibition that restricts virus release and thereby interferes with viral spread to new target cells. It also suggests that mammalian viruses may not be able to easily mutate to avoid tetherin because tetherin does not directly interact with any viral structural proteins. Sensitive viruses must therefore evolve specific ways of counteracting it. In the case of primate lentiviruses, several tetherin antagonists have now been identified. The Vpu accessory protein antagonizes tetherin in HIV-1 infected cells [5], [6], whereas in a variety of SIVs that do not encode a Vpu gene, the Nef protein can overcome the tetherin orthologues from their host species [33], [45]. Interestingly, HIV-2 [32] and at least one strain of SIV [39] have acquired the ability to antagonize tetherin with their envelope glycoproteins. Outside the *Retroviridae*, the ebolavirus glycoprotein has anti-tetherin activity [13] and here we propose that human herpesvirus KSHV antagonizes tetherin with K5.

While this study was in revision, a study from Mansouri and colleagues showed that tetherin could be degraded by K5 in an ubiquitin-dependent manner [46]. Our data demonstrate that K5 can fully substitute for Vpu in mediating the efficient release of HIV-1 particles from tetherin-expressing cells. This requires the K5 RING domain and leads to a cell-surface down-regulation of tetherin followed by its degradation in endosomal compartments. Down-regulation of tetherin by K5 and its targeting to endosomes requires the membrane proximal lysine residue K18 in the cytoplasmic tail, and this process is sensitive to inhibition of the proteasome with MG132. Since K5-induced ubiquitination of tetherin is dependent on its cytoplasmic tail lysines, the effects of proteasomal inhibition are likely to be due to depletion of cytoplasmic ubiquitin levels, rather than blocking proteasomal degradation of tetherin. Furthermore, K5-mediated tetherin degradation requires a functional ESCRT pathway as shown by the rescue of surface tetherin levels after expression of a dominant negative VPS4 protein. These observations suggest that K5 targets tetherin by inducing an ubiquitin-dependent sorting of tetherin to multivesicular bodies where it is destroyed in a lysozomal compartment, a similar mechanism to that of K3-mediated degradation of Class I MHC molecules [41]. K3 targets MHCI for ESCRT-dependent sorting and destruction via addition of a single ubiquitin moiety to the molecule's cytoplasmic tail through recruitment of the E2 enzymes UbcH5B and/or C [38]. Lysine-63 poly-ubiquitination is then induced through the subsequent recruitment of Ubc13 [38]. While we cannot rule out that K5 induces poly-ubiquitination of tetherin, we were only able to observe species consistent with mono-ubiquitination. Thus, while K5 induces ESCRT-dependent tetherin degradation, the precise molecular details of the endosomal targeting may differ between K3 and K5 targets. Whether endocytosis of tetherin is stimulated by K5, or whether it is routed to endosomes from the Golgi, bypassing the cell surface remains an interesting question. Our observation that surface down-regulation of tetherin is independent of its tyrosine-based endocytic-sorting signal [40] suggests the latter. Since K5 localizes mainly to the ER [47], tetherin ubiquitination could happen very early after synthesis leading to endosomal routing independently of the cell surface. Similarly, Mansouri and colleagues suggested that in K5-expressing cells, little tetherin reaches the PM, based on surface biotinylation experiments [46]. However, neither of these results is unambiguous, especially if cell surface turnover is fast. Further studies of the molecular details of K5 mechanism are required to fully dissect its effects on tetherin trafficking.

An important aspect of our study is the comparative analysis of the mechanisms by which Vpu and K5 achieve tetherin antagonism. Both proteins lead to cell surface down-regulation and degradation of tetherin but the mechanism of Vpu remains unclear. Several studies have shown that tetherin degradation is blocked by proteasomal inhibition [19], [34], [35], whereas others suggest endosomal degradation [36], [37]. It is clear that whilst Vpu-mutants that cannot interact with βTRCP2 cannot mediate tetherin degradation [36], [37], they can nonetheless antagonize tetherin and rescue viral release [17]. Thus, the SCF-Skp1-cullin 1 ubiquitin ligase complex and perhaps an ER-associated degradative pathway are implicated in tetherin degradation and this process presumably follows tetherin antagonism at the cell surface [35], [36]. Tetherin down-regulation and degradation might therefore not be as strictly linked during Vpu-mediated antagonism as it is during K5 mediated antagonism of tetherin. Furthermore, proteasomal inhibition appeared to be more potent than endosomal inhibition in rescuing cellular levels of tetherin in Vpu-expressing cells. Unlike K5, we saw no evidence of tetherin redistribution to endosomes in response to Vpu. Rather, residual tetherin can be seen in the TGN, consistent with a recent study suggesting that Vpu localization to the TGN is important for tetherin antagonism [48]. Similarly, proteasomal inhibition does not restore tetherin to the surface of Vpu-expressing cells, and neither does dominant negative VPS4 (not shown). Also consistent with these observations, is our observation that Vpu can induce ubiquitination of tetherin cytoplasmic tail lysine residues, but these are dispensable for Vpu sensitivity. Thus their ubiquitination appears to be a consequence of tetherin antagonism rather than the absolute requirement for K18 demonstrated for K5 sensitivity. In this respect, we suggest tetherin antagonism by Vpu precedes, and may not be dependent on, degradation, but rather results in the sequestration of tetherin away from budding virions, preventing incorporation. Thus there are more parallels with the mechanism by which HIV-2 and SIVtan envelopes antagonize tetherin through sequestration in TGN-associated compartments [32], [39]. K5's apparent inability to antagonize tetherin in 293T cells, cells that support Vpu's antagonism of tetherin, suggests that Vpu and K5 may require different cellular cofactors. Clearly, further comparative mechanistic studies will allow us to dissect the differences and similarities in the mode of action of these two very different proteins.

We, and others [46], have also shown that productive KSHV release is restricted by tetherin expression and knockdown of K5 expression imparted a block to virus release. Furthermore, tetherin expression is reduced on B cells after K5 expression. Importantly, this indicates that herpesvirus particle assembly is sensitive to the antiviral effects of tetherin. The mechanism of herpesvirus assembly and envelopment is complex and controversial, most studies have focused on herpes simplex virus type 1 (HSV-1). Immature HSV-1 capsids may bud through nuclear membrane, re-entering the cytoplasm, and then bud again into secretory vesicles [29] via an ESCRT dependent process [49]. Our data suggest that in the case of KSHV, at least one budding stage is through a membrane accessible to tetherin. Tetherin is highly expressed in terminally differentiated B cells and plasma cells, important cellular targets for KSHV [50]. B cell to plasma cell differentiation activates KSHV lytic replication through the activation of XBP-1 and the unfolded protein response [51]. Therefore the virus undergoes productive replication in cells that express high levels of tetherin. This cellular tropism may have provided the selective pressure for K5 evolution to target tetherin. K5 has the ability to modulate the expression of a variety of cell surface molecules involved in immuno-recognition (MHC and NK receptors) and cell adhesion, suggesting targeting of tetherin is part of a wider immuno-evasion strategy by KSHV [52]. Is tetherin antagonism found in other mammalian herpesviruses? B cell expression of tetherin would certainly suggest that this might be the case for Epstein Barr Virus (EBV). Removal of MHC and related molecules is certainly a common feature of several human herpesvirus immune evasion strategies [52], [53], [54], and it is likely that if other herpesviruses are sensitive to tetherin-mediated restriction, proteins with analogous function to K5 might also target tetherin. MARCH ligase homologues are also found in a variety of poxviruses [20], which also have highly complex envelopment strategies [55].

Aside from the cytoplasmic tail lysine residue K18, we do not yet fully understand the determinants of sensitivity for tetherin's antagonism by K5. Data presented herein show that while K5, like Vpu [18], [19], cannot target tetherin from Rhesus macaques, the TM domain positions that determine the difference in Vpu-sensitivity do not confer resistance to K5. Tetherin has been under high levels of positive selection during mammalian evolution, particularly in the cytoplasmic tail and TM domain, areas of the protein likely to be topologically accessible to viral antagonists [18], [19]. Several residues in the TM domain are responsible for the species-specific activity of Vpu. However, positive selection in other parts of tetherin may be due to selective pressure exerted by distinct viral countermeasures. We speculate that some mammalian herpesviruses, which show strong co-evolution with their hosts, may have contributed to this evolutionary pressure. Tetherin may therefore represent an extremely interesting case study in the evolution of an antiviral gene under regular assault by unrelated viral pathogens, as has been suggested for PKR [56].

In conclusion, here we have demonstrated that tetherin is capable of restricting a human herpesvirus and show that in the case of KSHV, the virus has co-opted an immuno-modulatory ubiquitin ligase to target this antiviral effector.

## Materials and Methods

### Plasmids, cells and antibodies

All adherent cells were maintained in Dulbecco's Modified Eagle Medium (DMEM) supplemented with 10% fetal calf serum and antibiotics. BCBL-1 cells were maintained in Roswell Park Memorial Institute (RPMI) medium supplemented with 10% FCS and antibiotics. HeLa, 293T and HT1080 cells were obtained from the ATCC. The HIV-1 indicator cell line, HeLa-TZM, that expresses HIV-1 receptors and an integrated HIV-1-LTR controlling expression of a beta-galactosidase reporter gene were kindly provided by John Kappes and Xiaoyun Wu via the NIH AIDS reagents program. HT1080/THN-HA is a clonal cell line expressing human tetherin with an internal HA tag inserted at nucleotide position 463 [18] expressed from an integrated MLV proviral vector, pLHCX (Clontech). Mutants of tetherin were constructed by standard methods and expressed from pCR3.1 and pLHCX. Vpu, K5 and a K5 mutant lacking amino acids 1–62 encompassing the RING domain (K5delRING) were amplified by PCR and inserted into pCR3.1 with C-terminal HA and mCherry fusion tags and the retroviral vectors pCxCR, which also encodes dsRED express as a marker gene, and pCMS28, a pMigR1 derivative encoding puromycin under control of an Internal Ribosome Entry Site (IRES). The molecular clones of HIV-1 NL4.3 and the Vpu-defective counterpart have been described previously [7]. Anti-HA monoclonal antibody HA1.11 was obtained from Covance, rabbit anti-HA polyclonal was obtained from Rockland and anti-BST2 monoclonal antibody was obtained from Abnova. Secondary Alexa Fluor 488, 594 and 633 antibodies for flow cytometry and microscopy were obtained from Molecular Probes. For quantitative western blotting, Licor 680 and 800nm secondary abs were used and blots scanned using a Licor scanner.

### Production of KSHV

HeLa cells infected with KSHV were made by infecting HeLa with rKSHV.219 a recombinant KSHV virus encoding RFP, GFP and puromycin resistance, a gift from Jeff Vieira [30]. Cells were selected and kept under puromycin selection. To induce rKSHV.219 into the lytic cycle, r219-Hela cells were seeded at 3.10^5^ cells/well onto 6-well plates and transfected with 1.5 µg of an expression factor for RTA (pCMV-RTA) (a gift from Adrian Whitehouse) using Fugene-6. RFP expression was visible a day after transfection. The transfection mix was removed and replaced with 2 ml of fresh medium. Virus was recovered another 24 hours later, i.e. 48 hours after transfection, and filtered through 0.45 µM device (Millipore) to remove any cellular debris. Infectious virus was measured by titration of supernatants onto 293T cells and total virus by QPCR on DNAse-I resistant genomes. To assay for KSHV release in the presence of tetherin, r219-Hela cells were transfected with pCMV-RTA and increasing doses of tetherin expression vector pCR3.1-THN [5]. Plasmid dose was kept constant using empty vector (pCDNA3.1). To assay for KSHV release under conditions where K5 expression is reduced, r219-Hela cells were seeded at 10^5^ cells per well in 6-well plates 24 hours prior to transduction with the lentiviral vectors encoding the K5 specific hairpins, or the empty vector, at a multiplicity of infection (MOI) of 5. 72 hours post-transduction, cells were counted, re-seeded into 6-well plates and transfected with RTA encoding plasmid as above.

### Quantitation of KSHV infectious particles by titration onto 293T

293T cells were seeded at 10^5^ cells/well in 12-well plates 24 hours prior to titration. For each viral collection 250 µl (a sixth) of the final volume of filtered virus was added to fresh medium to a final volume of 1 ml and used to infect 293T cells. Titrations were performed in duplicate using polybrene (4µg/ml). 12-well plates were subjected to spinoculation at 500 g for 1 hour at RT before being returned to the incubator. Cells were analyzed by flow cytometry 48 hours post-inoculation.

### Quantitation of KSHV genomes by Taqman PCR against KSHV early gene ORF37

KSHV genomes were quantified by extracting total DNA (QIAamp, QIAGEN) from DNAse-I treated supernatant (70 units/ml for 2 hours, RQ1 Promega, UK) with 40µg of salmon sperm DNA as a carrier (Sigma, Poole UK). 5µg of purified DNA was subjected to quantitative Taqman PCR for KSHV early gene ORF37 as described [57]. Absolute copy number was determined with reference to a standard curve derived by QPCR against serial dilutions of an ORF37 amplicon encoding plasmid, a gift from David Bibby and Duncan Clark, as described [58].

### Quantification of mRNA levels for the late gene ORF37 in r219-HeLa cell lysates by Q-RT-PCR

Total mRNA was extracted from r219-HeLa cells after virus collection, 48 hours post-RTA transfection (RNeasy, QIAGEN). cDNA syntheses were performed on 4 µl of the RNA (SuperScript II Reverse Transcriptase, Invitrogen) according to manufacturer's instructions. cDNAs were treated with 2 units of RNAse H (Invitrogen) for 20 minutes at 37°C before being used in taqman Q-PCR reactions for ORF37 and GAPDH. GAPDH primers were GAPDH forward primer, 5′-GGCTGAGAACGGGAAGCTT-3′; GAPDH reverse primer, 5′-AGGGATCTCGCTCCTGGAA-3′; GAPDH probe, 5′-FAM-TCATCAATGGAAATCCCATCACCA-TAMRA-3′. Absolute copy number was determined with reference to a standard curve derived by QPCR against serial dilutions of a GAPDH amplicon encoding plasmid. QPCR for ORF37 was performed as described above.

### Quantification of the number of episomes per nanogram of total DNA in r219-HeLa cells

Cellular DNA was extracted from r219-HeLa cells after virus collection, 48 hours post-RTA transfection (QIAamp, QIAGEN) and QPCR for ORF37 performed, as described above, on cellular associated episomes. Copy numbers were normalized to quantities of DNA used for each reaction.

### Production of retroviral vectors and infectious HIV-1 (VSV-G pseudotypes)

Semi-confluent 293T cells on 6-well dishes were transfected with 1µg of vector plasmid, 1µg of pMLVgag-pol or p8.91 (HIV-1 gag-pol, tat and rev expression vector) and 0.2µg pCMV-VSVG. For full length replication competent HIV-1 (VSV-G) pseudotypes, 293T cells were transfected with 2µg of pNL4.3 or pNL4.3(del Vpu) and 0.2µg of VSV-G. This leads to production of replication competent HIV-1 that has both VSV-G and HIV-1 gp160 envelope proteins on its surface. Virus and vector stocks were harvested 48h post-transfection. Those encoding florescent markers were titrated on HT1080 cells and analyzed by flow cytometry; lentiviral vectors encoding shRNA hairpins were titered by Q-PCR on 293T cells with primers specific for the HIV-1 LTR; and the endpoint titer of full length HIV-1 pseudotypes was determined on HeLa-TZM cells by staining infected foci with X-Gal 48h post-infection.

### HIV-1 release assays

Subconfluent HeLa cells were transfected with 500ng of HIV-1 proviral plasmid and 100ng of pCR3.1-Vpu or pCR3.1-K5-HA/K5delRING-HA using lipofectamine 2000 (Invitrogen). Viral supernatants and cell lysates were harvested 48h later. Supernatant virions were filtered and pelleted at 30000g through 20% sucrose/PBS cushion for 90 minutes. Lysates and virions were then separated by SDS-PAGE and HIV-1 Gag proteins detected using an anti-p24 monoclonal antibody CA183 (provided by B Chesboro through the NIH AIDS Reagents repository). In parallel, harvested supernatants were titered onto HeLa-TZM indicator cells and 48 hours later beta-gal activity was determined in cell lysates using a Tropix Beta-galactosidase activity kit (Molecular Probes). For assays involving stable HT1080/THN-HA cell lines, 10^5^ cells were plated per well in a 12 well plate and infected with 2×10^4^ infectious units (MOI 0.2) of HIV-1 (VSV-G) or HIV-1(del Vpu)(VSV-G) viral stocks. 48h later cell lysates and viral supernatants were treated as above.

### Flow cytometry and confocal microscopy

Cell surface staining for tetherin and THN-HA was performed using the appropriate antibodies by standard methods and analyzed on a FACS-Calibur (Becton-Dickinson). Cells for microscopy were grown on glass coverslips and transfected or treated with BafA1 (100nM), concanamycin A (100nM) or MG132 (1µg/ml) (Sigma, UK) for 10h. The cells were then fixed in 4% paraformaldehyde, permeabilized with 0.1% Triton-×100 and stained with the required primary and secondary antibody. Cells were then examined using a Zeiss confocal microscope.

### Tetherin ubiquitination assay

HeLa cells seeded on 10cm plates were transiently transfected with the indicated THN-HA expression vector (5ug) in combination with K5, Vpu or GFP (2µg) expression vectors and in the presence or absence of a 6-His-tagged ubiquitin encoding plasmid (2µg). 48h after transfection, cells were treated for 8h with BafA1 (Sigma) to prevent further tetherin degradation. Cells were lysed in 5M guanidinium hydrochloride, sonicated and ubiquitinated proteins were isolated by binding to 50µl of Ni2+ Nti-agarose (Invitrogen) for 3h at room temperature. The beads were eluted with 100mM imidazole. Lysates and pull-downs were then analyzed by western blot for THN-HA.

## Supporting Information

Figure S1 K5 does not antagonize tetherin-mediated restriction of HIV-1(del Vpu) in 293T cells. 293T cells were transfected with wild type HIV-1 or HIV-1(del Vpu) proviral plasmids in combination with either Vpu or K5 expression vectors and increasing doses of pCR3.1 THN-HA. 48h later supernatant infectivity was determined on HeLa-TZM cells and is shown plotted against tetherin input.
(0.26 MB EPS)

Figure S2 Vpu mediates the surface down-regulation of THN-HA but not THN-HA(delGI-T45I) or Rh-THN-HA in HT1080 cells. Surface expression levels of stably transduced HT1080 cells expressing THN-HA, THN-HA(delGI-T45I) or Rh-THN-HA and Vpu were measured by flow cytometry. The red line represents staining in unmodified HT1080 cells, the purple histogram represents tetherin expression levels in tetherin expressing cells and the green line represents tetherin levels after HIV-1 Vpu expression.
(0.29 MB EPS)
